# Zinc Binding by Histatin 5 Promotes Fungicidal Membrane Disruption in *C. albicans* and *C. glabrata*

**DOI:** 10.3390/jof6030124

**Published:** 2020-07-31

**Authors:** Hannah L. Norris, Rohitashw Kumar, Chih Yean Ong, Ding Xu, Mira Edgerton

**Affiliations:** Department of Oral Biology, School of Dental Medicine, University at Buffalo, Foster Hall Buffalo, NY 14214, USA; hlnorris@buffalo.edu (H.L.N.); rohitash@buffalo.edu (R.K.); chihyean@buffalo.edu (C.Y.O.); dingxu@buffalo.edu (D.X.)

**Keywords:** *Candida albicans*, *Candida glabrata*, Histatin 5, P113, zinc, membrane disruption

## Abstract

Histatin 5 (Hst 5) is an antimicrobial peptide produced in human saliva with antifungal activity for opportunistic pathogen *Candida albicans*. Hst 5 binds to multiple cations including dimerization-inducing zinc (Zn^2+^), although the function of this capability is incompletely understood. Hst 5 is taken up by *C. albicans* and acts on intracellular targets under metal-free conditions; however, Zn^2+^ is abundant in saliva and may functionally affect Hst 5. We hypothesized that Zn^2+^ binding would induce membrane-disrupting pores through dimerization. Through the use of Hst 5 and two derivatives, P113 (AA 4-15 of Hst 5) and Hst 5ΔMB (AA 1-3 and 15-19 mutated to Glu), we determined that Zn^2+^ significantly increases killing activity of Hst 5 and P113 for both *C. albicans* and *Candida glabrata*. Cell association assays determined that Zn^2+^ did not impact initial surface binding by the peptides, but Zn^2+^ did decrease cell association due to active peptide uptake. ATP efflux assays with Zn^2+^ suggested rapid membrane permeabilization by Hst 5 and P113 and that Zn^2+^ affinity correlates to higher membrane disruption ability. High-performance liquid chromatography (HPLC) showed that the higher relative Zn^2+^ affinity of Hst 5 likely promotes dimerization. Together, these results suggest peptide assembly into fungicidal pore structures in the presence of Zn^2+^, representing a novel mechanism of action that has exciting potential to expand the list of Hst 5-susceptible pathogens.

## 1. Introduction

Histatin 5 (Hst 5) is one of multiple Histatin peptides found in the oral environment of humans and higher primates [1,2]. Hst 5 is notable for its high antimicrobial activity against the oral opportunistic pathogen *Candida albicans* [2] and other *Candida* species [3,4]. The full mechanism of action remains unclear, but multiple cellular effects are known, including efflux of small molecules like potassium, magnesium [5], ATP [6], ROS generation [7], and cell cycle arrest [8]. At the same time, it was discovered that Hst 5 binds to multiple divalent cations including copper and zinc [9], potentially with physiological relevance [10], though the function of this capability is still poorly understood [11,12,13,14,15,16].

The effects of Zn^2+^ binding on the conformation, oligomerization state, and membrane interactions of the Hst 5 peptide have been described [11,14,15], but this has also raised questions about the practical application of Zn^2+^ to alter or improve fungicidal activity. Though there are studies on the effect of both copper [12] and iron [16] on Hst 5 killing activity, comparatively little is known about the direct effect of Zn^2+^ on Hst 5 efficacy or the mechanism involved. Hst 5 kills fungal cells through multiple intracellular mechanisms under established in vitro experimental conditions in which there is a requirement for Hst 5 to be actively taken up by fungal polyamine transporters Dur3 and Dur31 [17]. Exogenous expression of these transporters in the naturally Hst 5-resistant *Candida glabrata* results in significantly increased killing activity, which has further substantiated Hst 5 as having an intracellular mechanism of action against fungi [18]. This mechanism is unusual because other cationic antimicrobial peptides (AMPs) that are biochemically similar to Hst 5 function predominantly by pore formation and act directly upon microbial membranes [19]. In fact, although active uptake of Hst 5 is required to achieve efficient antifungal activity under in vitro conditions, Hst 5 binds as well to model membranes as true membrane translocating peptides [20] and causes visible, though mild membrane defects in *C. albicans* [21]. Despite the ability of Hst 5 to interact with fungal membranes, the peptide is only capable of lethally disrupting a small proportion (10%) of *C. albicans* membranes at high concentrations [22] or when remaining on the cell surface over time [23]. Thus, the ability of Hst 5 to associate with membranes is almost entirely separate from membrane disruption. Although Hst 5 appears to have little ability to disrupt membranes in low salt buffers (i.e. 10 mM sodium phosphate buffer (NaPB)), introduction of other divalent ions such as Zn^2+^ may alter the functional properties of Hst 5 in the oral environment. 

Saliva is the functional environment for Hst 5 and contains measurable levels of numerous metal cations [24]. We found that Zn^2+^ is the most abundant metal in the saliva of healthy adults among other salivary metals, including Cu, Fe, Ni, and Mn [24]. Zinc concentrations in healthy adult saliva range from 0–3.77 µM [24], though this does not include fluctuations that may occur from diet or Zn^2+^ supplementation [25,26]. Concentrations of Hst 5 in whole saliva range from 0.7–5.6 µM [27]. Based upon these studies, an average physiological ratio of salivary Zn^2+^: Hst 5 is less than one Zn^2+^ per peptide. However, very little work has been done to study how Hst 5 activity is modulated at this physiological ratio [10,13,16]. 

A review by Łoboda et al. (2018) [28] highlights the diversity of bactericidal and fungicidal membrane binding and pore forming peptides and proteins potentiated by metal binding [28]. Zinc in particular has been implicated in enhancing Hst 5 bacterial and model membrane interactions [13,15], and there are numerous other examples of Zn^2+^-enhanced protein- and peptide-membrane interactions in eukaryotic and prokaryotic cells [29,30,31]. Besides this, multiple factors highlight the possibility that addition of Zn^2+^ might increase membrane disruption capabilities compared to Hst 5 alone. Hst 5 is capable of catalyzing vesicle fusion in the presence of Zn^2+^ [10], indicating direct binding with membrane phospholipids and the ability to affect membrane structure. Importantly, Zn^2+^ binding stabilizes Hst 5 in bioactive conformations [11] and also promotes dimerization [14]. Peptide dimerization and aggregation have a complex relationship with AMP activity [32,33] but are highly potentiating for some pore-forming peptides [34,35,36], and pre-assembly of oligomeric peptide structures can speed up pore formation [34,37,38]. Furthermore, Zn^2+^ increases the surface adsorption capabilities of Hst 5 at a broad pH range [15], which may lead to general membrane disruption by increasing the number of molecules at the membrane and carpeting the cell surface with high charges [39]. Though our previous work has established that Hst 5 functions by disrupting intracellular ion balance, these studies were carried out without Zn^2+^ and at relatively high doses of added Hst 5. In this study, we examine Hst 5 at a much lower dose per fungal cell, with added Zn^2+^ at a physiological ratio.

To understand the role of peptide length and sequence in the effects of Zn^2+^ binding, we utilized Hst 5 and two derivative peptides, including a Hst 5 proteolytic product P113 and a metal-binding mutant Hst 5ΔMB, which lack canonical ATCUN and HExxH metal-binding motifs. P113 is often referred to as the “active” fragment of Hst 5 because it retains all of the candidacidal activity of the full-length Hst 5 peptide [40]. A direct comparison of Zn^2+^ affinity between Hst 5 and P113 has never been carried out, but we expected P113 to have lower Zn^2+^ affinity than Hst 5 due to the lower number of Zn^2+^-binding residues. Through a comparison of these three peptides, we show the importance of Zn^2+^ affinity and dimerization to a novel Zn^2+^-mediated membrane-disruption mechanism of fungicidal activity for Hst 5. This is the first study to show that Zn^2+^ binding significantly potentiates Hst 5 and P113 fungicidal activity, and furthermore, it is the first study linking Zn^2+^ binding to in vitro evidence of membrane disruption in *C. albicans*.

## 2. Materials and Methods

### 2.1. Yeast Strain, Media, and Reagent Preparation

Experiments were carried out with *Candida albicans* strain SC5314 [41] or *Candida glabrata* strain Cg931010 [42]. Liquid and solid pre-mixed yeast-peptone-dextrose media was used to grow yeast cells (YPD and YPD-Agar, respectively) (Fisher Scientific, Waltham, MA, USA). All media was prepared in deionized water with 50 µg/ mL supplemented uridine (Sigma Aldrich, St. Louis, MO, USA), and sterilized by autoclave. Metal salt solutions of ZnSO_4_·7H_2_O (Fisher Scientific, Waltham, MA, USA) were made in High-performance liquid chromatography (HPLC)-grade water (JT Baker, Phillipsburg, NJ, USA) and used within one day of preparation. Here, 10 mM sodium phosphate buffer, pH 7.4 (NaPB) (Fisher Scientific, Waltham, MA, USA) was prepared directly in HPLC water containers (JT Baker, Phillipsburg, NJ, USA) to prevent metal ion contamination, and filter sterilized. Zincon monosodium salt dye (Sigma Aldrich, St. Louis, MO, USA) for metal titrations was prepared in 100% methanol (Fisher Scientific, Waltham, MA, USA). Briefly, cell culture conditions were as follows for *C. albicans* and *C. glabrata* in all experiments: Liquid cell cultures were inoculated from a single colony and grown overnight (30 °C, 220 rpm). Cultures were then diluted to OD_600_ of 0.3–0.4 and re-cultured to OD_600_ = 0.9–1.0. Cells were then washed twice in NaPB and diluted for use in experiments. For candidacidal assays, a cell concentration of 3.4 × 10^7^ cells/ mL was used for a dosage of 0.44 fmol peptide/cell, and a cell concentration of 1.0 × 10^6^ cells/ mL was used for all other dosages. Uptake assays and ATP assays were both performed with a cell concentration of 3.4 × 10^7^ cells/ mL.

### 2.2. Peptides

Three peptides—Histatin 5, P113, and Histatin 5ΔMB—were used in this study. Hst 5 is 24 amino acids long (DSHAKRHHGYKRKFHEKHHSHRGY), while the proteolytic product P113 is only 12 AA (AKRHHGYKRKFH) but retains similar killing efficacy. As a control, we designed a metal binding mutant of Hst 5 (Hst 5 ΔMB, QQQAKRHHGYKRKFQQQQQSHRGY) that replaces both the N-terminal ATCUN motif (AA 1-3) and the canonical HExxH motif (AA 15-19) of Hst 5 with glutamines. Though we were primarily interested in the effects of Zn^2+^ rather than copper in this study, we chose to mutate both metal binding sites for the purpose of comparing and contrasting activity and function of Hst 5ΔMB and P113. All three peptides were synthetically manufactured by Genemed Synthesis, INC. (San Antonio, Texas, USA) and dissolved in RNase and DNase free water (Corning, Corning, NY, USA). Master stocks for long term storage were maintained at −80 °C, while diluted working stock solutions were stored at −20 °C, which were thawed as needed and kept at 4 °C to minimize peptide precipitation from repeated freeze-thaw cycles. Peptide solution concentrations were confirmed by NanoDrop (ThermoFisher Scientific, Waltham, MA, USA).

### 2.3. Peptide Solubility Assay

Aggregation was quantified by measuring peptide precipitation from NaPB. Molar ratios of [0:0], [0:1], [1:2], [1:1], [2:1], [3:1], and [4:1] ZnSO_4_ to 15 µM peptide were used and samples were incubated for 30 min at room temperature (20 °C) before centrifugation. Samples were spun at 20000× *g* for 10 min at room temperature and the protein concentrations of the supernatants were subsequently measured using microvolume spectrophotometry (NanoDrop One, Thermofisher Scientific, Waltham, MA, USA) Data were cleaned using robust regression and outlier removal (ROUT) with Q set to 1%. Cleaned data were baseline corrected by subtracting peptide- and metal- free NaPB (0:0). Total moles of peptide per sample (1:2, 1:1, 2:1, 3:1, 4:1) were subtracted from the corresponding Zn^2+^-free controls (0:1) to provide moles lost value. Percent aggregation out of total peptide added was calculated. Curves were fit using a second order polynomial. Statistical differences between the peptides at each metal salt titration were calculated using two-way ANOVA with Tukey’s multiple comparison test.

### 2.4. Zinc-Binding Competition Assay

Binding competition assays were carried out utilizing the colorimetric copper and Zn^2+^ binding dye Zincon. Peptides (20 µM) were mixed with Zincon at a 1:1 ratio and titrated with increasing equivalents of ZnSO_4_. Absorbance at 621 nm was measured at each addition of metal salt using a Lambda 25 UV/Vis spectrophotometer (Perkin Elmer, Waltham, MA, USA). Data were curve fit using a one site competition equation.

### 2.5. High-Performance Liquid Chromatography

To determine the oligomerization state of Hst 5, Hst 5ΔMB, and P113, experiments were performed using an Aglient 1260 Infinity modular HPLC system (Agilent Technologies Inc., Santa Clara, CA, USA) and a Zenix SEC-80 column (Sepax Technologies, Inc., Newark, DE, USA). The mobile phase used was 10 mM NaPB, pH 7.4, with or without 164.67 µM ZnSO_4_. Hst 5, Hst 5ΔMB, or P113 at 329.34 µM were prepared in 10 mM NaPB and allowed to come to room temperature before loading. Samples were run at a flow rate of 1 mL/min for 15 min, and protein peaks were measured using absorbance at 210 nm. Analysis of HPLC traces were performed using OpenLab software (Agilent Technologies Inc., Santa Clara, CA, USA). Peak identification and peak baselines were determined manually using visual comparison to known standards. Subsequently, statistical differences in the percentage of monomer, dimer, and higher-order peaks of peptide alone and added Zn^2+^ traces were compared in GraphPad Prism (GraphPad Software Inc., San Diego, CA, USA) using two-way ANOVA and Tukey’s multiple comparisons test. Representative traces were subjected to second-order smoothing. 

### 2.6. Candidacidal Assays

Cells (*C. albicans* or *C. glabrata*) were cultured as previously described [43]. Briefly, cells were cultured in YPD to OD_600_ = 1.0 (30 °C, 220 rpm), then washed in NaPB, and added to peptides alone or peptides pre-incubated with Zn^2+^ in NaPB at a dosage of 0.44, 3.75, 7.50, 15, or 30 fmol peptide/cell. After incubation (30 °C, 220 rpm), cells were then diluted, plated, and grown for at least 18 h on YPD-agar to determine viability. Experiments were repeated on at least three different days. Significance of differences in killing between Zn^2+^ and Zn^2+^-free samples were calculated using one-way ANOVA with Sidak’s multiple comparisons test.

### 2.7. Peptide Uptake Assay

*C. albicans* cells were added to pre-incubated peptides (30 min, 20 °C) alone or at a 1 Zn^2+^:2 peptide ratio, and additionally to Hst 5 at a 3 Zn^2+^:2 peptide ratio, using a dose of 0.44 fmol peptide/cell. After cells were added, samples were vortexed briefly and immediately centrifuged for 2 min at 6000× *g* at 20 °C, after which 10 µL supernatant was removed to new tubes and stored at 4 °C for quantification. Pelleted cells were resuspended with vortexing and incubated at 30 °C with shaking at 220 rpm. Samples were removed from 30 °C after intervals of 10, 15, and 20 min total incubation time for centrifugation and supernatant removal as described above. Protein concentration in supernatant samples was measured by NanoDrop. Experiments were repeated on at least three different days. The initial time point was taken as a measure of surface binding, and was subtracted from 10, 15, and 20-min time points to determine uptake. Linear regressions were performed on uptake values. Significance of differences in uptake rate and binding between zinc added and zinc free samples were calculated using two-way ANOVA with Sidak’s multiple comparisons test.

### 2.8. Extracellular ATP Quantification

*Candida albicans* (SC5314) or *Candida glabrata* (Cg931010) cells were prepared as for peptide uptake assays, with the exception that cells were added to samples at a dosage of 0.88 fmol peptide/cell in a 1:2 ratio of ZnSO_4_:peptide. After samples were mixed, each was immediately centrifuged for 2 min at 6000× *g* at room temperature, after which 10 µL supernatant was removed to new tubes and added to 84 µL boiling TE buffer (50 mM Tris pH 7.5, 2 mM EDTA pH 8) (1-min time point). Pelleted cells were resuspended with vortexing. After this, samples were incubated at 30 °C (220 rpm) and removed from incubation for a 10-min time point. Samples in TE buffer were boiled for two more minutes and stored on ice for quantitation. Then, 25µL of each sample were added to 10 µL Staybrite adenosine triphosphate (ATP) assay mix and 65 µL assay buffer (Biovision, Milpitas, CA, USA), and luminescence was immediately measured using a FlexStation 3 plate reader (Molecular Devices, San Jose, CA, USA). Experiments were carried out on at least three separate days. Data were interpolated using a standard curve to determine moles of ATP in each sample. Significant differences between Zn^2+^ added and Zn^2+^-free samples at each time point were determined using one-way ANOVA and Sidak’s multiple comparisons test.

### 2.9. Statistical Analysis

All calculations and statistical analysis were performed using GraphPad Prism 7.04 (GraphPad Software, San Diego, CA, USA).

## 3. Results

### 3.1. Zinc Binding Characteristics of Hst 5, P113, and Hst 5ΔMB 

**Figure 1:**
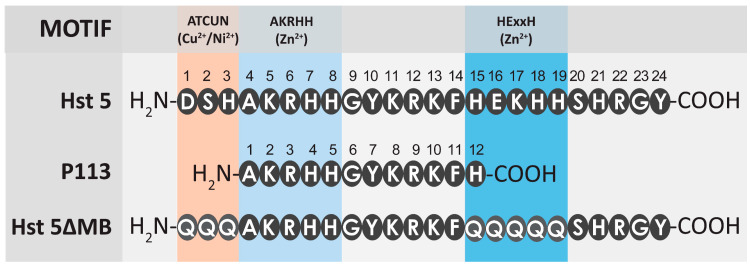
A comparison of the length and metal binding regions of amino acid sequences for Hst 5, Hst 5ΔMB, and P113. Three metal binding motifs are labeled—ATCUN (Cu^2+^, orange), HExxH (Zn^2+^, blue), and (H)AKRHH (Zn^2+^, light blue). P113 is truncated to amino acids 4-15 of Hst 5. The ATCUN (AA 1-3) and HExxH (AA 15-19) motifs of Hst 5ΔMB are mutated to glutamines.

Hst 5 is comprised of 24 amino acid residues with multiple overlapping metal binding motifs including a Cu^2+^ binding ATCUN motif [12] and two discrete Zn^2+^ binding motifs including a canonical HExxH motif and another Zn^2+^-affinity sequence closer to the N-terminus, (H)AKRHH [14]. (Figure 1). The P113 proteolytic product of Hst 5 is 12 amino acids in length and spans amino acids 4–15 of Hst 5 [40]. P113 also has some metal binding ability due to a lower affinity Zn^2+^ binding site despite lacking the HExxH motif [44] and most of the Cu^2+^ binding ATCUN motif [45]. Our designed peptide Hst 5ΔMB is the same length as Hst 5 but we expected that the mutant peptide and P113 would both have decreased Zn^2+^ binding compared to Hst 5 as both contain only one Zn^2+^ binding motif (AKRHH).

**Figure 2:**
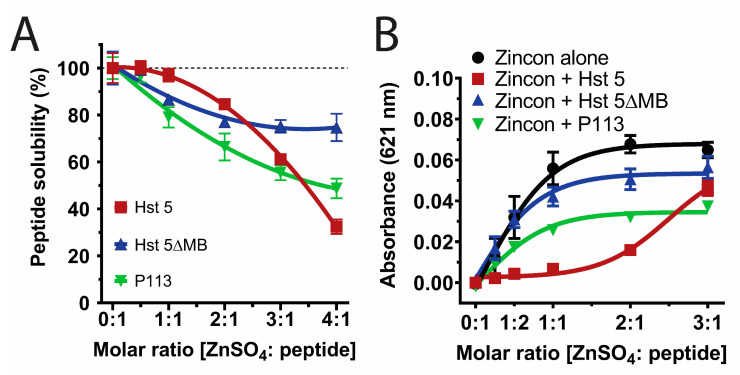
The three Hsts are all soluble at a physiological ratio of Zn^2+^ to peptide but differ in relative Zn^2+^ affinity. (**A**) Percent solubility of 15 µM Hst 5 (red), Hst 5ΔMB (blue), and P113 (green) in 10 mM sodium phosphate buffer pH 7.4 with increasing ratio of Zn^2+^ incubated at room temperature for 30 min. Peptide solubility was measured via NanoDrop A205 after removal of insoluble aggregates by centrifugation. (**B**) Binding competition assay with Zincon and peptides at 20 µM in 10 mM sodium phosphate buffer pH 7.4 to establish relative affinity of Hst 5 (red), Hst 5ΔMB (blue), and P113 (green) for Zn^2+^ at increasing molar ratios. Absorbance of Zincon at 621 nm was measured as an inverse indicator of relative peptide affinity, as Zincon has a blue color when bound to zinc. Experiments were performed in triplicate and averaged.

Since Zn^2+^ binding strongly affects Hst 5 solubility [46], we compared solubility of these peptides in 10 mM NaPB by varying the molar ratio of Zn^2+^ added to Hst 5 (red), Hst 5ΔMB (blue), or P113 (green) at Zn^2+^ to peptide ratios from 0:1 to 4:1 (Figure 2A). All three peptides were completely soluble with one Zn^2+^ to two peptides. However, a 1:1 ratio resulted in 3% precipitation of Hst 5, 14% of Hst 5ΔMB, and 21% precipitation of P113, and a 4:1 ratio further caused 68%, 25%, and 51% precipitation of Hst 5, Hst 5ΔMB, and P113, respectively. Therefore, a ratio of 1 Zn^2+^ to 2 peptides was chosen to maintain full solubility of all peptides in subsequent experiments. Also, this ratio most closely represents physiological ratios of Hsts with Zn^2+^ levels in saliva [24,27].

The relative binding affinity of each peptide for Zn^2+^ was measured by its ability to compete for Zn^2+^ with indicator dye Zincon, which has a high absorbance value at 621 nm when Zn^2+^ bound and low absorbance when not Zn^2+^ bound (Figure 2B). Zincon + Hst 5ΔMB (blue line) was not significantly different from Zincon alone (black line), confirming that Hst 5ΔMB had the lowest affinity for Zn^2+^ among the tested peptides. In contrast, Hst 5 (red line) competed Zn^2+^ away from Zincon up to a ratio of two Zn^2+^ to one peptide before binding sites were saturated and excess Zn^2+^ remained in solution (indicated by a large increase in absorbance at three Zn^2+^ to one peptide). This agrees with previous findings that Hst 5 contains two higher-affinity Zn^2+^ binding sites [47]. It is interesting to note that the majority of Hst 5 precipitation occurred after both high affinity zinc binding sites were filled, and coincided with an increase in Zincon absorbance, indicating that fully saturated zinc binding decreased peptide solubility. P113 (green line) had intermediate Zn^2+^ affinity relative to Hst 5 and Hst 5ΔMB, and P113 competed Zn^2+^ away from Zincon only at a molar ratio of 1:1 or less. These results indicate that Hst 5 has two Zn^2+^ binding sites and P113 has one, while Hst 5ΔMB has only low affinity for Zn^2+^ despite containing one Zn^2+^ binding motif. This also indicates that the Zn^2+^ binding motif present in Hst 5ΔMB and P113 has variable affinity for Zn^2+^ dependent upon the surrounding amino acids.

### 3.2. Hst 5ΔMB C. albicans Killing is Reduced Accompanying Mutation of one Zn^2+^ Binding Site While Hst 5 Killing Is Increased in the Presence of Zn^2+^

**Figure 3:**
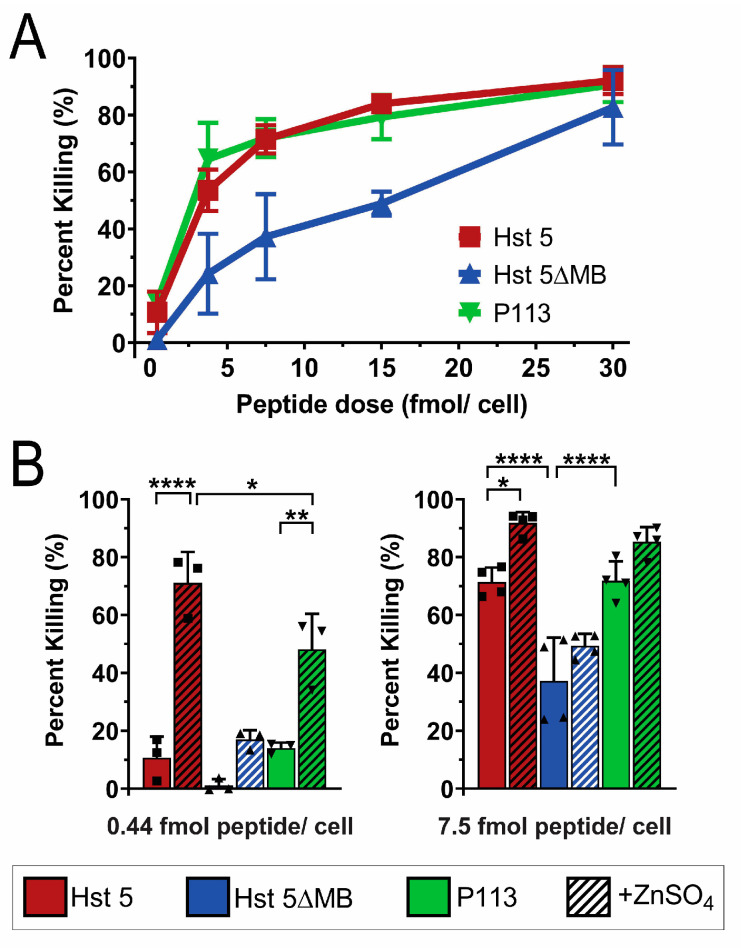
Hst 5ΔMB has less killing activity than Hst 5 or P113 and does not have increased killing activity with added Zn^2+^. (**A**) Candidacidal assays of *C. albicans* SC5314 cells with 1 h incubation at 30 °C of Hst 5 (red), Hst 5ΔMB (blue), and P113 (green) at dosages of 0.44, 3.75, 7.5, 15, and 30 fmol/cell in 10 mM sodium phosphate buffer pH 7.4. Data represent at least two replicates for each dose. (**B**) Candidacidal assays of *C. albicans* SC5314 cells with 1 h incubation at 30 °C of Hst 5 (red), Hst 5ΔMB (blue), and P113 (green) with and without a 1:2 ratio of Zn^2+^: peptide (hatched) at dosages of 0.44 fmol/cell (left) and 7.5 fmol/cell (right) in 10 mM sodium phosphate buffer pH 7.4. Squares and triangles indicate individual replicates. Significance of differences in killing activity were calculated using one way ANOVA with Sidak’s multiple comparisons test. * indicates *p* ≤ 0.05, ** indicates *p* ≤ 0.01, **** indicates *p* ≤ 0.0001.

We first tested the fungicidal activity of Hst 5, P113 and Hst 5ΔMB in standard buffer (10 mM NaPB free of trace metals) to determine the importance of the intact ATCUN and HExxH motifs in killing (Figure 3). We tested candidacidal activity of the three peptides over a range of doses (0.44, 3.75, 7.5, 15 and 30 fmol/ cell) following 1 h incubation (Figure 3A). Hst 5 and P113 had similar killing activity over the entire range of doses tested achieving 90% killing at 30 fmol/cell. Hst 5ΔMB had decreased killing activity compared to Hst 5 and P113 at most doses, up to 80% killing at a dose of 30 fmol/ cell. 

Next we examined how the presence of zinc altered killing activity (Figure 3B). To determine the importance of Zn^2+^ binding sites for functional activity of the peptides, Zn^2+^ was added to the buffer at a ratio of 1 Zn^2+^ to 2 peptides at the 0.44 and 7.5 fmol/cell doses. Strikingly, the presence of Zn^2+^ significantly increased killing of Hst 5 from 10 to 70% at 0.44 fmol/cell and P113 from 10% to 50% but did not significantly alter the killing activity of Hst 5ΔMB. A similar elevation of killing with Zn^2+^ was found for Hst 5 at 7.5 fmol/cell, but the relative increase was less due to higher basal killing. These results correlate with the relative Zn^2+^ binding affinity among the three peptides. The relatively low Zn^2+^ binding affinity of Hst 5ΔMB was associated with no increase in killing activity, the moderate relative affinity of P113 for Zn^2+^ with a significant increase in killing activity, while the highest affinity Zn^2+^ binding by Hst 5 was associated with even greater killing activity with added Zn^2+^.

**Figure 4:**
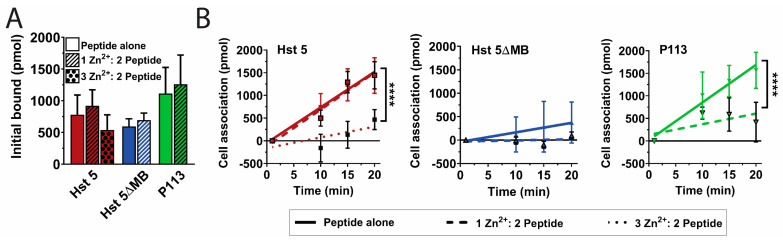
Zinc decreases uptake of P113 at a one Zn^2+^ to two peptides ratio and Hst 5 at a three Zn^2+^ to two peptides ratio. Cell association assays were performed at 30 °C with 0.44 fmol/cell. Hst 5 (red), Hst 5ΔMB (blue), and P113 (green) in 10 mM sodium phosphate buffer pH 7.4 with or without added Zn^2+^ at a one Zn^2+^ to two peptides ratio (hatched) or a three Zn^2+^ to two peptides ratio (dots). Time points were taken at 1, 10, 15, and 20 m. (**A**) One minute time points were taken as initial binding and (**B**) cell association attributed to active uptake was calculated by subtracting the initial time point from the 10, 15, and 20 m time points. Linear regressions were performed to determine rate of uptake. Experiments were repeated on at least three separate days and averaged. Significance of differences in initial binding were calculated using one-way ANOVA with Sidak’s multiple comparisons test. Significance of differences in rate of uptake were calculated using one-way ANOVA with Sidak’s multiple comparisons test. **** indicates *p* ≤ 0.0001

Since Hst 5 killing is most highly associated with its intracellular uptake [17], we hypothesized that introduction of Zn^2+^ would alter the Hst 5 secondary or tertiary structure to permit more rapid uptake by *C. albicans* cells. We therefore performed Hst-cell-association assays to determine the relative surface binding and uptake of the three peptides with or without Zn^2+^ (Figure 4). A pre-incubation time point (1 min) was taken to determine initial surface binding of the three peptides, which ranged from 600 to 1100 pmol out of 3000 pmol total added, but differences were not significant and addition of Zn^2+^ did not change the relative initial binding of peptide to the cell (Figure 4A). Unexpectedly, addition of Zn^2+^ to Hst 5 at a 1:2 ratio (dashed red line) did not significantly alter total uptake or rate of uptake compared to Hst 5 alone (solid red line) (Figure 4B). Hst 5 was the only peptide to retain relatively high uptake with a 1:2 ratio of Zn^2+^ to peptide, so we also tested a higher molar ratio of Zn^2+^ to ascertain if Zn^2+^ binding at a second site decreased Hst 5 uptake. The addition of higher levels of Zn^2+^ at a 3:2 ratio (dotted red line) reduced the rate and total uptake of Hst 5 to 470 pmol. As expected from its low killing activity, Hst 5ΔMB (solid blue line) had a total uptake of only 370 pmols, which was further decreased to 90 pmol (dashed blue line) by the addition of Zn^2+^. The total uptake of P113 alone (green line) reached 1570 pmol which was significantly (*p* ≤ 0.001) reduced to 420 pmol with the addition of Zn^2+^ (dashed green line). Thus, the increased killing activity of Hst 5 and P113 in the presence of Zn^2+^ was not a result of increased peptide binding or uptake.

**Figure 5:**
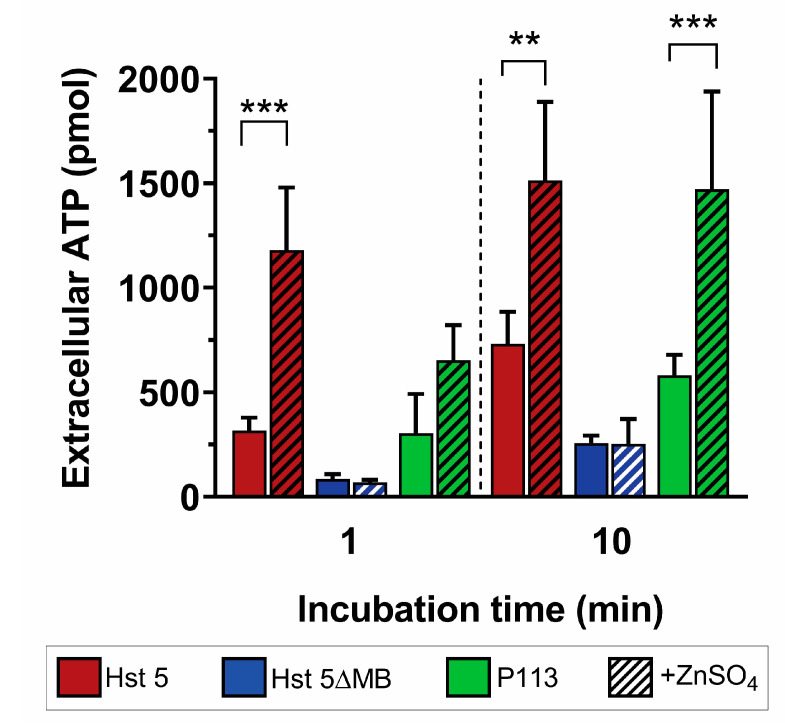
Zinc induces almost immediate ATP efflux. *C. albicans* SC5314 bioluminescent ATP efflux assay. Cells were treated at 30 °C with 0.88 fmol/ cell. Hst 5 (red), Hst 5ΔMB (blue), or P113 (green) in 10 mM sodium phosphate buffer pH 7.4 with or without added Zn^2+^ at a one to two peptides ratio (hatched). Cells were removed from samples via centrifugation and aliquots of supernatant were taken at 1 min and 10 min time points to measure extracellular ATP by luminescence. Experiments were repeated on at least three separate days and averaged. Significance of differences in ATP efflux calculated using one way ANOVA with Sidak’s multiple comparison test. ** indicates *p* ≤ 0.01, *** indicates *p* ≤ 0.001.

We next assessed whether the increase in Zn^2+^-mediated killing activity of Hst 5 and P113 was associated with ATP efflux, a classic indicator of membrane disruption by AMPs [21]. ATP efflux was measured for each peptide with or without Zn^2+^ after 1 min and 10 min of incubation with *C. albicans* (Figure 5). We found that Hst 5 (red) induced a total ATP efflux of 317 pmol at 1 min, which increased to 732 pmol after a 10 min incubation. Addition of Zn^2+^ (red hatched) significantly increased ATP release to 1180 pmol at 1 min and 1513 pmol at 10 min, representing a 3.7-fold increase in ATP efflux at 1 min and 2.1-fold at 10 min. Hst 5ΔMB alone (blue) initiated ATP efflux of 85 and 257 pmols at 1 and 10 min that did not significantly increase with added Zn^2+^ (blue hatched). For P113 alone (green), ATP efflux was 304 and 582 pmols at 1 and 10 min, while the addition of Zn^2+^ (green hatched) did not increase release at 1 min but significantly increased ATP release to 1471 pmol (2.5-fold) at 10 min. These results show that Hst 5 or P113 alone induce some release of ATP from cells, but the addition of Zn^2+^ significantly elevated ATP efflux suggesting membrane permeabilization of *C. albicans* cells. 

**Figure 6:**
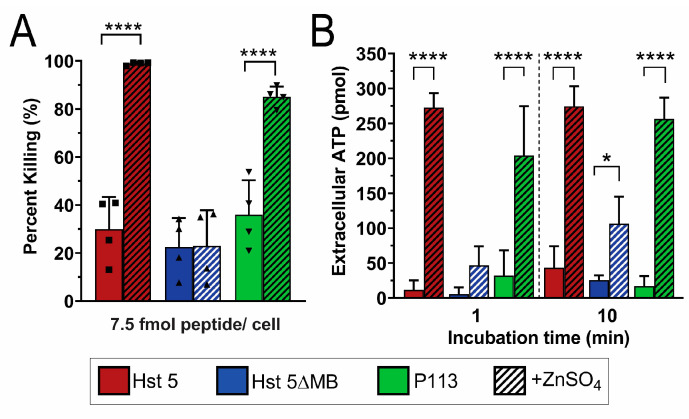
Zinc greatly increases Hst 5 and P113 killing of *C. glabrata*, and induces almost immediate ATP efflux. *C. glabrata* Cg931010 cells were used in candidacidal assays and ATP efflux assays. (**A**) For candidacidal assays, cells were incubated for 1 h at 30 °C with Hst 5 (red), Hst 5ΔMB (blue), and P113 (green) with and without a 1:2 ratio of Zn^2+^ to peptides (hatched) at 7.5 fmol peptides/cell in 10 mM sodium phosphate buffer pH 7.4. Squares and triangles indicate individual replicates. (**B**) In bioluminescent ATP efflux assays, cells were treated with 0.88 fmol/cell Hst 5 (red), Hst 5ΔMB (blue), or P113 (green) in 10 mM sodium phosphate buffer pH 7.4 with or without added Zn^2+^ at a one to two peptides ratio (hatched). Cells were removed from samples via centrifugation and aliquots of supernatant were taken at 1 and 10 min time points to measure extracellular ATP by luminescence. Both experiments were repeated on at least three separate days and averaged. Significance of differences in killing and ATP efflux were calculated using one way ANOVA with Sidak’s multiple comparison test. * indicates *p* ≤ 0.05, **** indicates *p* ≤ 0.0001

Since Zn^2+^ appeared to initiate fungicidal activity by membrane disruption, we examined whether Zn^2+^ could function in a similar manner against *C. glabrata* that does not transport Hst 5 intracellularly and consequently is insensitive to Hst 5. We therefore performed killing (Figure 6A) and ATP efflux assays (Figure 6B) in *C. glabrata* with peptides with and without added Zn^2+^. We found that a 7.5 fmol/cell dose of Hst 5 (red) induced 30% killing, which is 40% less than in *C. albicans* under the same conditions, while the addition of Zn^2+^ (red hatched) increased killing to nearly 100%. Hst 5ΔMB (blue) only produced about 20% killing with or without added Zn^2+^, but P113 (green) increased from about 40% to 80% killing with the addition of Zn^2+^. As suggested by the low killing activity of Hst 5, ATP efflux was minimal in *C. glabrata* when treated with peptide alone. Hst 5 induced 10 pmol ATP efflux total at 1 min which increased to 40 pmol after 10 min. However, when Zn^2+^ was added, ATP efflux increased to 270 pmol at one min, which did not further increase after 10 min, indicating that maximal ATP efflux occurred almost immediately. Interestingly, although ATP efflux due to Hst 5ΔMB alone never exceeded 30 pmol total, when Zn^2+^ was added, a significant increase to over 100 pmol occurred by 10 min. This indicates that Hst 5ΔMB is also minimally capable of membrane disruption, though to a lesser extent than Hst 5. P113 also had very low ATP efflux like Hst 5 or Hst 5ΔMB, but when Zn^2+^ was added, an immediate increase in ATP efflux was apparent, reaching 200 pmol at 1 min and almost 260 pmol at 10 min.

**Figure 7:**
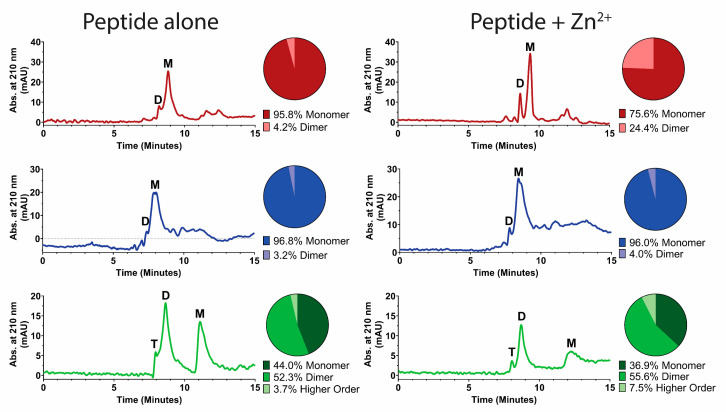
Zinc binding by Hst 5 promotes zinc-induced soluble dimers. Size-exclusion high-performance liquid chromatography (HPLC) was performed with protein detection at A210 to determine dimerization state of (top, red) Hst 5, (middle, blue) Hst 5ΔMB, and (bottom, green) P113 at a concentration of 329.34 µM in mobile phase composed of either zinc-free 10 mM sodium phosphate buffer pH 7.4, or buffer with added zinc at a concentration of 164.67 µM. Each condition was run in triplicate. Peak identification was made based upon comparison to known standards and area under the curve of peaks was used to calculate the relative percentage of peptide in monomer (M), dimer (D), and higher order oligomer (T) form (circle graphs). Representative traces of individual runs were subjected to second order smoothing for visualization.

We hypothesized that peptide-membrane-disruption effects induced by Zn^2+^ may be partly a result of Zn^2+^-induced peptide dimerization, since dimerization is dependent on the number and location of histidines [12] and has previously been correlated to fusogenic activity [10]. Therefore, peptide dimerization induced by Zn^2+^ was examined using size-exclusion HPLC (Figure 7). Hst 5 (red trace) eluted in two peaks—one at 8 min that we interpreted as dimer formation and a second larger peak at 9 min—that represented the majority of the peptide and was assigned as the monomeric fraction. The addition of Zn^2+^ to the mobile phase resulted in a distinguishable change of the relative ratio between the monomeric and dimeric peaks. Analysis of peak areas found that addition of Zn^2+^ increased the percentage of peptide in dimer form from 4% to 24%. For Hst 5ΔMB (blue trace), the majority of peptide eluted as a monomer peak at 8 min, and the addition of Zn^2+^ to the mobile phase did not change the elution profile. P113 (green trace) eluted as three peaks—the smallest at 8 min, a larger peak at 9 min, and another larger peak at 11.5 min—corresponding to tetrameric, dimeric, and monomeric fractions by molecular weight. Interestingly, this shows that a large proportion (about 50%) of P113 naturally forms dimers in 10 mM NaPB (pH 7.4). The addition of Zn^2+^ to the mobile phase slightly increased the percentage of dimeric and oligomeric species of P113. Thus, Zn^2+^ induces dimer formation in Hst 5, but not Hst 5ΔMB, and somewhat increases dimeric and higher order structures in P113.

## 4. Discussion

We found that Zn^2+^ binding to Hst 5 and P113 significantly increased their ability to kill *C. albicans* concurrent with rapid ATP release suggesting membrane disruption. In step with this finding, we discovered that Zn^2+^ binding potentiates the candidacidal activity of Hst 5 and P113 for *C. glabrata* by a similar mechanism, which is of exciting clinical relevance because *C. glabrata* is highly resistant to Hst 5 [18] and other AMPs [48]. This work is also of significance in understanding the role of Hst 5 in the oral environment, as we found an average physiological ratio of Hst 5 and Zn^2+^ (1 Zn^2+^:2 peptides) potentiates the candidacidal activity of Hst 5 against these two *Candida* spp, suggesting that Zn^2+^ binding is relevant to the function of Hst 5 in typical levels of Zn^2+^ found in saliva [24].

Of the three classic models of membrane disruption by antimicrobial peptides—carpet, barrel stave, and toroidal as reviewed in Łoboda et al (2018) [28]—our data suggests that Hst 5 most likely functions in the toroidal model. Hst 5 binding to *C. albicans* was not significantly changed by the addition of Zn^2+^, suggesting that Hst 5 does not function through non-specific carpet disruption. Barrel-stave pore-forming peptides require a hydrophobic character to insert into a membrane. Hst 5 is highly charged and lacks the hydrophobicity necessary to insert directly into a membrane to form a typical barrel-stave pore [19]. Toroidal pores are formed by more polar peptides that bind to lipid head groups and induce curvature until pores develop, consistent with Hst 5 membrane disruption having a toroidal mechanism.

**Figure 8:**
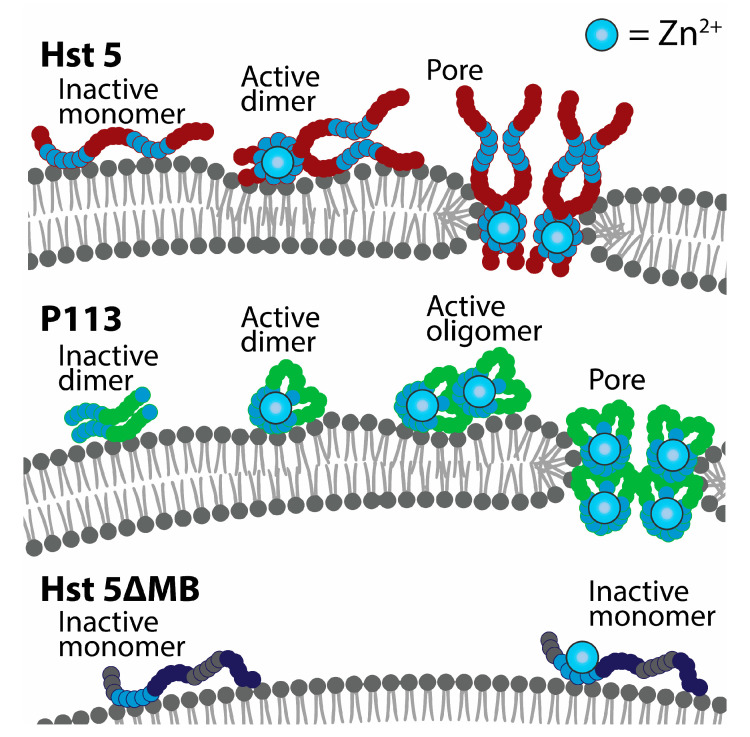
A model of Zn^2+^-induced pore formation by Hst 5 and P113. Zinc binding induces soluble dimers of Hst 5 that bind to the cell surface of *C. albicans* and *C. glabrata*. Interaction with fungal membranes induces dimer units to assemble into higher order structures, which induces membrane curvature and toroidal pores. P113 functions similarly but is dimeric before Zn^2+^ binding and requires more assembly steps to form a pore structure of the same size as Hst 5. Hst 5ΔMB binds to zinc but does not form dimers or assemble into higher order structures when membrane bound.

We also showed that Hst 5 dimerizes at a low 1:2 ratio of Zn^2+^ to peptides. This agrees with the historical view that the canonical Zn^2+^-binding HExxH motif facilitates Zn^2+^-bridged dimerization interactions [10] and recent work showing dimerization experimentally [14]. Dimerization of Hst 5 in solution could be an important precursor to membrane disruption, as this is a mechanism that can speed up pore formation after membrane binding [32,34]. However, Hst 5ΔMB does not dimerize in solution with added Zn^2+^ but does induce ATP efflux in *C. glabrata*, suggesting that Zn^2+^ binding without soluble dimerization can slightly increase membrane stress. Additionally, P113 naturally forms dimers but did not show any indication of membrane disruption without the addition of Zn^2+^, suggesting that pore-forming peptide–peptide interactions at the cell membrane are specifically mediated by Zn^2+^ binding. A model of Zn^2+^-induced peptide self-assembly at the membrane accounts for the both delayed membrane disruption by P113 seen in our ATP assays in *C. albicans,* and the slight increase in ATP efflux in *C. glabrata* due to Hst 5ΔMB with added Zn^2+^. First, pore-forming peptides have length requirements and must span the width of a membrane in order to generate a pore [49]. P113 binds Zn^2+^ with less relative affinity and is half the length of Hst 5, so it may be less prone to pore assembly at the membrane. In our proposed model, P113 must go through more Zn^2+^-induced aggregation steps than Hst 5 in order to form a pore because it is too short to span the membrane and therefore requires twice the number of molecules to form a pore the same size as Hst 5 (Figure 8). In contrast, we propose that Hst 5ΔMB undergoes slow and reduced aggregation due to lower Zn^2+^ affinity and minimal soluble dimerization so that membrane effects are much less lethal to cells.

Cytosolic transport is an important determinant of *C. albicans* susceptibility to killing by Hst 5 [17]. We found that the molar binding ratio of Zn^2+^ with Hst 5 profoundly affected active peptide uptake (Figure 4), suggesting that dual Zn^2+^ binding plays a role in fine tuning the effect and targets of Hst 5. We previously established that P113 requires a specific amino acid sequence for cytosolic transport by *C. albicans* cells [50], and later this was proposed to be three amino acids, Lys-Phe-His (KFH) that make up AA 10–12 for P113 or 13–15 for Hst 5 [51]. The Zn^2+^ coordinating site of P113 is known and includes part of the KFH sequence [44], which suggests that Zn^2+^ binding could interfere with intracellular transport. Indeed, we found that uptake of P113 was lost with the addition of a 1:2 molar ratio of Zn^2+^:peptide. Similarly, Hst 5ΔMB, in which KFH has been mutated to KFQ, has low uptake regardless of the addition of Zn^2+^. In light of this, it is significant that Hst 5 does not have decreased uptake at a molar ratio of 1 Zn^2+^ to 2 peptides, but uptake was decreased when three Zn^2+^ per two peptides was used. This suggests that Zn^2+^ does not first bind at the KFH sequence, but the second bound Zn^2+^ blocks KFH to reduce uptake. This effect is not related to the dimerization of Hst 5 or P113, since P113 is 50% dimer with or without added Zn^2+^, and Hst 5 has good uptake at a ratio of Zn^2+^ with significant dimer formation.

We speculate that transport of Zn^2+^-bound Hst 5 into the cytosol is a functional mechanism to maximize killing activity. While it is generally accepted that salivary Hst 5 functions in innate immunity for oral opportunistic pathogens [52], not all fungal species have susceptibility to intracellularly targeted Hst 5. Increasing the molar binding ratio of Zn^2+^ to peptide modulates the target of Hst 5 from intracellular to the membrane, but there is an ideal ratio with maximal solubility, killing activity, and dimer formation where both targets can be acted on simultaneously. This is important because differences in ATP efflux and killing activity indicate that *C. albicans* and *C. glabrata* are not equally susceptible to membrane disruption by Hst 5, which is likely due to variability in cell wall or membrane components. Dual Zn^2+^ binding allows Hst 5 to act on the separate fungicidal targets of two oral pathogens with differing susceptibilities under the same conditions, suggesting that Hst 5 could also be involved in the control of other fungal and bacterial pathogens regardless of uptake ability. Further work is needed to elucidate the full scope of the role of Hst 5 in oral health in combination with salivary Zn^2+^.

## 5. Conclusions

In this study, we determined a new mode of action for Hst 5 that is modulated by zinc binding at a physiological molar ratio of Zn^2+^:peptide. This finding could have important implications in our understanding of the innate immune mechanisms in the oral environment, and furthermore provides valuable information to clinicians about the effect of Zn^2+^ on Hst 5 and derivatives like P113 that might have clinical relevance in treatment of oral fungal infections. This work highlights that Zn^2+^ ions have a functional antifungal role in the complex oral environment.
